# Supplementary material for: Clinical Findings and Antimicrobial Susceptibility of Anaerobic Bacteria Isolated in Bloodstream Infections
Source: Antibiotics (Basel). 2020 Jun 19;9(6):345. doi: 10.3390/antibiotics9060345 (PMC7345846; doi:10.3390/antibiotics9060345)
Supplement: Supplementary file 1 [file antibiotics-09-00345-s001.pdf]

Table S1. Resistance rate (%) of anaerobic bacteria against selected antimicrobial agents obtained from bacteraemias.

	Number	BEN	MET	MOX	IMI	AMC	CLI	PIT
Bacteroides spp.	**62**	98.3	3.2	35.4	3.2	32.2	45.1	14.5
Clostridium spp.	**34**	8.8	11.7	8.8	0	2	2.5	2.9
Fusobacterium spp.	**10**	10	0	10	0	0	20	0
Prevotella spp.	**3**	66.6	0	0	0	33.3	100	0
GPACs	**22**	4.7	4.7	23.8	0	0	54.5	4.7
Other GPABs	**9**	44.4	22.2	22.2	0	0	11.1	11.1
Veillonella	**1**	100	0	0	0	0	0	100

BEN: benzylpenicillin; **MET:** metronidazole; **MOX:** moxifloxacin; **IMI:** imipenem; **MER:** meropenem; **AMC:** amoxicillin-clavulanate; **CLI:** clindamycin;

PIT: piperacillin-tazobactam; **VAN:** vancomycin; **GPACs:** Gram-positive anaerobic cocci; **GPABs:** Gram-positive anaerobic bacilli
